# Is early childhood development impeded by the birth timing of the younger sibling?

**DOI:** 10.1371/journal.pone.0268325

**Published:** 2022-05-10

**Authors:** Gursimran Dhamrait, Melissa O’Donnell, Hayley Christian, Gavin Pereira

**Affiliations:** 1 Telethon Kids Institute, The University of Western Australia, Perth, Western Australia, Australia; 2 School of Population and Global Health, The University of Western Australia, Perth, Western Australia, Australia; 3 School of Health and Society, The University of Wollongong, Wollongong, New South Wales, Australia; 4 Australian Centre for Child Protection, University of South Australia, Adelaide, South Australia, Australia; 5 Curtin School of Population Health, Curtin University, Perth, Australia; 6 Centre for Fertility and Health (CeFH), Norwegian Institute of Public Health, Oslo, Norway; 7 enAble Institute, Curtin University, Perth, Western Australia, Australia; Pontificia Universidad Catolica de Chile, CHILE

## Abstract

**Background:**

This study investigated whether the timing of birth of the younger siblings was associated with the risk of the older siblings’ developmental vulnerability in early childhood.

**Methods:**

Linkage of population-level birth registration, hospital, and perinatal datasets to Australian Early Development Census (AEDC) records (2009–2015), enabled follow-up of a cohort of 32,324 Western Australia born singletons. Children with scores <10^th^ percentile on an individual AEDC domain (Physical Health and Wellbeing; Social Competence; Emotional Maturity; Language and Cognitive Skills (school-based); and Communication Skills and General Knowledge) were classified as developmentally vulnerable. Modified Poisson Regression was used to estimate relative risks (RR) for associations between post-birth interpregnancy intervals (IPIs) and developmental vulnerability.

**Results:**

Relative to post-birth IPIs of 18–23 months, post-birth IPIs of <6 and 6–11 months were associated with an increased risk of children being classified as DV1 (aRR 1.21, 95% CI: 1.11–1.31) and DV2 (aRR 1.31, 95% CI: 1.15–1.49); and DV1 (aRR 1.10, 95% CI: 1.03–1.17) and DV2 (aRR 1.21, 95% CI: 1.09–1.34), respectively. Post-birth IPIs of <6 months were associated with an increased risk on four of the five AEDC domains. Post-birth IPIs of 48–60 months were associated with an increased risk of developmental vulnerability; however, the risk was statistically significant for DV1, DV2 and the domains of Emotional Maturity and Language and Cognitive Skills (school-based).

**Conclusions:**

Developmental vulnerability was associated with having a closely spaced younger sibling (<12 months post-birth IPIs). Optimising birth spacing should be further investigated as a potential means for improving child development outcomes.

## Introduction

Given the relatively short birth intervals and relatively long dependency periods of human offspring, there is an increased need to understand the impact that siblings may have on each other’s learning and development [1, 2]. Studies have often replaced measures of birth spacing (sibship density) with the number of siblings (hereafter sibship size) [3] or birth order, due to either data availability or birth spacing being inappropriately measured [3, 4]. The inverse relationship between sibship size and educational outcomes and school performance is a consistent finding throughout status attainment literature; however, there are limited studies examining birth spacing effects on child development outcomes [5].

One of the major hypotheses for examining the effects of increasing sibship size on child outcomes is *the resource dilution hypothesis*, which states that a family’s resources are finite, and thus, children’s cognitive, educational, emotional, and physical development will be compromised with increasing sibling numbers [6]. Furthermore, lower socioeconomic status families have been reported to have an increased number of children compared to socioeconomically advantaged families, thereby exacerbating the effects of resource dilution [7]. Thus, in cases where the spacing between siblings is relatively short, the older sibling has access to familial resources for a shorter duration of time. This may impact the older child’s developmental outcomes when compared to children who have longer spacing between them and their subsequent sibling and as a result, have access to total familial resources for a greater period.

*The confluence model* [8] is linked with the resource dilution hypothesis and suggests that the intellectual development of a child is dependent on the intellectual environment in which they grow. Given that intellectual growth is a function of age, the intellectual milieu, the average intellectual levels of all members in a child’s family, is relatively high for the first-born child but decreases with increasing sibling numbers [5]. The theory underpinning this model could be extended to hypothesise that children with closely born subsequent siblings will experience an earlier decline in the quality of their intellectual environment and, thus, may be at a greater risk for developmental vulnerabilities when compared to children with longer intervals between them and their subsequent sibling.

Alternatively, the confluence model also hypothesises a *teaching-function effect* whereby the youngest child (and an only child) will not benefit from the opportunity to teach younger siblings [9]. Thus, the *no-one-to-teach hypothesis* postulates that this pedagogic experience stimulates the intellectual development of the older child and assists in the development of learning skills for the younger child [9, 10]. Together, these theoretical models feed into the *resource augmentation theory*, which suggests that sibship size is advantageous as older siblings can take the role of an attachment figure for younger siblings, thereby reducing competition between siblings [11, 12]. Although resource augmentation translates to hypothesised benefits that the younger sibling receives, as older siblings have the ability to increase total household resources [12], younger siblings may also have the ability to contribute to total household resources. The presence of younger siblings can provide an opportunity for bi-directional development whereby siblings can develop skills in perspective-taking, emotional understanding, negotiation and persuasion, and problem-solving [13, 14]. Thus, younger siblings can also influence the development of competencies, including emotional maturity and social competence in the older sibling [14, 15]. Older children with closely spaced siblings may be more likely to have peer-like relationships rather than enact the role of an attachment figure for their siblings [16]. Thus, older siblings with closely spaced siblings may benefit to a greater degree from inter-sibling interactions than children with widely spaced siblings, as the nature of the sibling relationships is likely to be different.

This study aimed to examine the relationship between birth spacing (measured as post-birth interpregnancy intervals (IPIs)) and early childhood developmental vulnerability of older siblings in a large sample of Australian children.

## Methods

### Data sources

Anonymised individual-level records from the Australian Early Development Census (AEDC) were obtained for all available years (2009, 2012, and 2015). Perinatal and birth-related records were obtained from the Midwives Notification System to identify sibling births and derive post-birth IPIs. Western Australian Register for Developmental Anomalies (WARDA) records were used to identify children with a diagnosed developmental disability. Data linkage was conducted independently of the researchers by the Western Australian (WA) Data Linkage Branch.

### Study population

**Fig 1:**
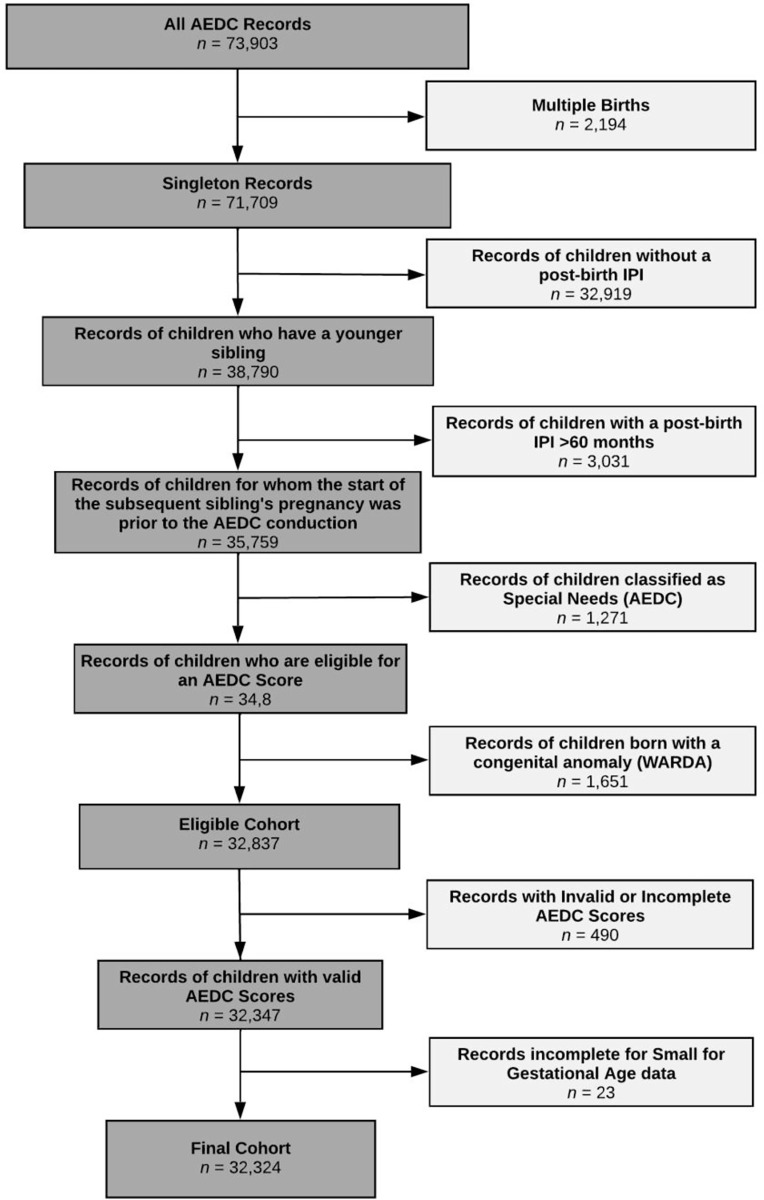
Eligible cohort and numbers included for analyses. AEDC = Australian Early Development Census. WARDA = Western Australian Register of Developmental Anomalies. IPI = Interpregnancy Interval.

The study population comprised of all children born in WA with an AEDC record in either 2009, 2012, or 2015 (*n* = 73,903). Records of children were sequentially excluded from the study if they, i) were from a multiple birth (*n* = 2,194); ii) did not have a post-birth IPI (i.e. children who are an only child or last-born) (*n* = 32,919); iii) had a post-birth IPI >60 months (i.e. the start of the subsequent pregnancy was after the AEDC was conducted on the cohort child) (*n* = 3,031); iv) were identified by their teacher as having ‘special-needs’ based on a clinically diagnosed physical or intellectual disability (*n* = 1,271); v) were reported as having any congenital anomaly in WARDA (*n* = 1,651); vi) had either an incomplete or missing AEDC score (*n* = 490); or vii) had missing small for gestational age data (*n* = 23). The final study cohort consisted of 32,324 children (Fig 1).

### Exposure variables

Post-birth IPI was derived as the time between the birth of the index child and the start of pregnancy of their next youngest sibling (i.e., date of birth of the succeeding child minus the gestational age of the succeeding child). In line with IPI studies [17–21], short post-birth IPIs were classified as; <6, 6–11, and 12–17 months. Long post-birth IPIs were classified as 24–35, 36–47, and 48–60 months. A post-birth IPI of 18–23 months was the reference category for this study.

### Outcome measure

The AEDC is a national census of early childhood development spanning five developmental domains, i) Physical Health and Wellbeing; ii) Social Competence; iii) Emotional Maturity; iv) Language and Cognitive Skills (school-based), and v) Communication Skills and General Knowledge. The AEDC is conducted every three years, with the first national data collection conducted in 2009 [22]. In Western Australia, this means that children must turn five years by the middle of the year of completion of the AEDC. Children who score <10^th^ percentile for a given domain are classified as ‘developmentally vulnerable’ for that domain. AEDC cut-off scores are based on the first national collection, and the 2009 cut-off scores apply to all subsequent collections [23]. In this study, two summarised outcome measures were used: developmentally vulnerable on one or more AEDC domains (DV1) and developmentally vulnerable on two or more AEDC domains (DV2), and assessed developmental vulnerability on each AEDC domain.

### Adjustment variables

Adjustment variables were selected on the basis of both availability of data and the findings of previous studies (Table 1) [24–26]. We obtained child, pregnancy and birth, and sociodemographic characteristics specific to the cohort child from the linked data sources, including sex of the cohort child, age at AEDC competition, maternal smoking status during pregnancy, preterm birth, small for gestational age, parity, maternal age at the time of child’s birth, child speaks a language other than English at home, ethnicity [27] of the child, preschool attendance, maternal marital status at the time of child’s birth, maternal and paternal occupation at birth, [28] the Accessibility and Remoteness Index of Australia (ARIA) [29] and the Index of Relative Socioeconomic Disadvantage (IRSD) [30].

**Table 1:** Sociodemographic characteristics of the study cohort.

Characteristics		Post-Birth Interpregnancy Interval						
		*n =* 32,324						
		<6	6–11	12–17	18–23	24–35	36–47	48–60
		*n =* 1561	*n =* 5333	*n =* 7008	*n =* 5623	*n =* 6870	*n =* 3737	*n =* 2192
**Child**								
Sex of Child	Male	759 (48.6)	2714 (50.9)	3468 (49.5)	2858 (50.8)	3549 (51.7)	1911 (51.1)	1105 (50.4)
	Female^a^	802 (51.4)	2619 (49.1)	3540 (50.5)	2765 (49.2)	3321 (48.3)	1826 (48.9)	1087 (49.6)
Age Category at Time of AEDC Completion^b^ (years)	1	262 (16.8)	916 (17.2)	1217 (17.4)	976 (17.4)	1189 (17.3)	677 (18.1)	380 (17.3)
	2^a^	1177 (75.4)	3951 (74.1)	5178 (73.9)	4208 (74.8)	5141 (74.8)	2747 (73.5)	1618 (73.8)
	3	122 (7.8)	466 (8.7)	613 (8.7)	439 (7.8)	540 (7.9)	313 (8.4)	194 (8.9)
**Pregnancy and Birth**								
Maternal Smoking Status during pregnancy	No^a^	1069 (68.5)	4497 (84.3)	6149 (87.7)	4880 (86.8)	5919 (86.2)	3089 (82.7)	1767 (80.6)
	Smoked	492 (31.5)	836 (15.7)	859 (12.3)	743 (13.2)	951 (13.8)	648 (17.3)	425 (19.4)
Preterm birth	≥37 weeks^a^	1434 (91.9)	5011 (94.0)	6627 (94.6)	5272 (93.8)	6426 (93.5)	3491 (93.4)	2037 (92.9)
	<37 weeks	127 (8.1)	322 (6.0)	381 (5.4)	351 (6.2)	444 (6.5)	246 (6.6)	155 (7.1)
Small for gestational age	No^a^	1409 (90.3)	4888 (91.7)	6430 (91.8)	5172 (92.0)	6312 (91.9)	3416 (91.4)	1978 (90.2)
	Yes	152 (9.7)	445 (8.3)	578 (8.2)	451 (8.0)	558 (8.1)	321 (8.6)	214 (9.8)
Parity	First Birth^a^	772 (49.5)	3297 (61.8)	4651 (66.4)	3642 (64.8)	4151 (60.4)	2031 (54.3)	1114 (50.8)
	Second Birth	388 (24.9)	1209 (22.7)	1476 (21.1)	1295 (23.0)	1835 (26.7)	1143 (30.6)	712 (32.5)
	Third Birth	209 (13.4)	488 (9.2)	506 (7.2)	423 (7.5)	525 (7.6)	364 (9.7)	228 (10.4)
	Fourth Birth	84 (5.4)	175 (3.3)	204 (2.9)	136 (2.4)	206 (3.0)	125 (3.3)	87 (4.0)
	≥Fifth Birth	108 (6.9)	164 (3.1)	171 (2.4)	127 (2.3)	153 (2.2)	74 (2.0)	51 (2.3)
Maternal Age at time of Child’s Birth (years)	<20	170 (10.9)	317 (5.9)	348 (5.0)	314 (5.6)	442 (6.4)	343 (9.2)	241 (11.0)
	20–24	438 (28.1)	1130 (21.2)	1259 (18.0)	1026 (18.2)	1416 (20.6)	905 (24.2)	598 (27.3)
	25–29^a^	348 (22.3)	1608 (30.2)	2282 (32.6)	1860 (33.1)	2109 (30.7)	1016 (27.2)	518 (23.6)
	30–34	127 (8.1)	574 (10.8)	753 (10.7)	500 (8.9)	583 (8.5)	237 (6.3)	109 (5.0)
	35–39	458 (29.3)	1662 (31.2)	2322 (33.1)	1886 (33.5)	2280 (33.2)	1227 (32.8)	720 (32.8)
	≥40	20 (1.3)	42 (0.8)	44 (0.6)	37 (0.7)	40 (0.6)	9 (0.2)	6 (0.3)
**Sociodemographic**								
Language other than English Spoken at Home by Child	No^a^	1371 (87.8)	4757 (89.2)	6346 (90.6)	5099 (90.7)	6039 (87.9)	3209 (85.9)	1857 (84.7)
	Yes	190 (12.2)	576 (10.8)	662 (9.4)	524 (9.3)	831 (12.1)	528 (14.1)	335 (15.3)
Ethnicity of Child	Non-Indigenous^a^	1311 (84)	4886 (91.6)	6570 (93.8)	5267 (93.7)	6399 (93.1)	3410 (91.2)	1957 (89.3)
	Indigenous Australian	250 (16.0)	447 (8.4)	438 (6.3)	356 (6.3)	471 (6.9)	327 (8.8)	235 (10.7)
Attended Preschool	No	134 (8.6)	375 (7.0)	487 (6.9)	376 (6.7)	411 (6.0)	247 (6.6)	147 (6.7)
	Yes^a^	1328 (85.1)	4700 (88.1)	6257 (89.3)	5053 (89.9)	6183 (90.0)	3336 (89.3)	1939 (88.5)
	Unavailable	99 (6.3)	258 (4.8)	264 (3.8)	194 (3.5)	276 (4.0)	154 (4.1)	106 (4.8)
Maternal Marital status at Time of Child’s Birth	Married (inc. de facto) ^a^	1321 (84.6)	4797 (89.9)	6405 (91.4)	5120 (91.1)	6159 (89.7)	3229 (86.4)	1854 (84.6)
	All other	232 (14.9)	495 (9.3)	553 (7.9)	452 (8.0)	655 (9.5)	483 (12.9)	329 (15.0)
	Unavailable	8 (0.5)	41 (0.8)	50 (0.7)	51 (0.9)	56 (0.8)	25 (0.7)	9 (0.4)
Maternal Occupation Status^c^	0-<20 (lowest status)	447 (28.6)	993 (18.6)	1069 (15.3)	832 (14.8)	1114 (16.2)	771 (20.6)	517 (23.6)
	≥20-<40	414 (26.5)	1293 (24.2)	1616 (23.1)	1336 (23.8)	1769 (25.7)	1036 (27.7)	633 (28.9)
	≥40-<60	296 (19.0)	1189 (22.3)	1671 (23.8)	1362 (24.2)	1678 (24.4)	845 (22.6)	476 (21.7)
	≥60-<80	103 (6.6)	489 (9.2)	730 (10.4)	647 (11.5)	659 (9.6)	306 (8.2)	143 (6.5)
	≥80–100^a^ (highest status)	126 (8.1)	1040 (19.5)	1559 (22.2)	1178 (20.9)	1273 (18.5)	514 (13.8)	241 (11.0)
	Unavailable	175 (11.2)	329 (6.2)	363 (5.2)	268 (4.8)	377 (5.5)	265 (7.1)	182 (8.3)
Paternal Occupation Status^c^	0-<20 (lowest status)	201 (12.9)	466 (8.7)	498 (7.1)	429 (7.6)	589 (8.6)	354 (9.5)	250 (11.4)
	≥20-<40	731 (46.8)	2296 (43.1)	2888 (41.2)	2282 (40.6)	2878 (41.9)	1615 (43.2)	1005 (45.8)
	≥40-<60	151 (9.7)	641 (12.0)	928 (13.2)	802 (14.3)	924 (13.4)	534 (14.3)	265 (12.1)
	≥60-<80	168 (10.8)	865 (16.2)	1173 (16.7)	931 (16.6)	1140 (16.6)	514 (13.8)	293 (13.4)
	≥80–100^a^ (highest status)	111 (7.1)	708 (13.3)	1109 (15.8)	878 (15.6)	915 (13.3)	423 (11.3)	179 (8.2)
	Unavailable	199 (12.7)	357 (6.7)	412 (5.9)	301 (5.4)	424 (6.2)	297 (7.9)	200 (9.1)
Accessibility and Remoteness Index of Australia^d^	1 (Least Remote)^a^	1022 (65.5)	3631 (68.1)	4914 (70.1)	3983 (70.8)	4883 (71.1)	2628 (70.3)	1519 (69.3)
	2	207 (13.3)	591 (11.1)	757 (10.8)	586 (10.4)	717 (10.4)	380 (10.2)	214 (9.8)
	3	183 (11.7)	603 (11.3)	704 (10.0)	563 (10.0)	668 (9.7)	375 (10.0)	225 (10.3)
	4	83 (5.3)	262 (4.9)	354 (5.1)	290 (5.2)	324 (4.7)	184 (4.9)	116 (5.3)
	5 (Most Remote)	46 (2.9)	146 (2.7)	173 (2.5)	118 (2.1)	187 (2.7)	119 (3.2)	85 (3.9)
	Unavailable	20 (1.3)	100 (1.9)	106 (1.5)	83 (1.5)	91 (1.3)	51 (1.4)	33 (1.5)
Index of Relative Socioeconomic Disadvantage^e^	1 (Most Disadvantaged)	446 (28.6)	1029 (19.3)	1113 (15.9)	904 (16.1)	1202 (17.5)	757 (20.3)	473 (21.6)
	2	354 (22.7)	968 (18.2)	1228 (17.5)	959 (17.1)	1198 (17.4)	744 (19.9)	483 (22.0)
	3	286 (18.3)	1007 (18.9)	1324 (18.9)	1043 (18.5)	1366 (19.9)	718 (19.2)	412 (18.8)
	4	259 (16.6)	1121 (21)	1618 (23.1)	1315 (23.4)	1505 (21.9)	804 (21.5)	400 (18.2)
	5 (Least Disadvantaged)^a^	202 (12.9)	1083 (20.3)	1598 (22.8)	1320 (23.5)	1508 (22)	670 (17.9)	403 (18.4)
	Unavailable	14 (0.9)	125 (2.3)	127 (1.8)	82 (1.5)	91 (1.3)	44 (1.2)	21 (1.0)

^a^Reference group for regression analysis.

^b^Age categories classified as; 1) ≥3 years 10 months to <5 years and one month, 2) ≥5 years and one month to <5 years and 10 months, 3) ≤5 years and 10 months.

^c^Maternal and Paternal Occupation Status are classified into five categories in line with the Australian Socioeconomic Index 2006 (AUSEI06); low AUSEI06 values represent low-status occupations.

^d^Categorised as nationally defined into five classes of remoteness; 1 = Major Cities of Australia (least remote) to 5 = Very Remote Australia (most remote).

^e^Categorised as nationally defined quintiles (1 = most disadvantaged to 5 = least disadvantaged); as quintiles are defined nationally (rather than within study population), numbers within each category vary from 20% of the total.

## Multiple imputation

Overall, complete covariate information was available for 84.3% (*n =* 27,265) of the study population. To minimise bias attributable to missing data [31], multivariate imputation by chained equations, using 20 imputed datasets, was applied, and the adjusted analyses presented were performed by pooling estimates from these imputed datasets.

## Sensitivity analysis

To assess the sensitivity of our results to imputation, we compared the main results, which were based on the imputed data (*n =* 32,324), to the results based on the analysis of the complete cases only (*n =* 27,265; S1 Table).

### Statistical modelling

Modified Poisson regression with robust error variance [32, 33] was used to estimate the relative risk of children being classified as DV1, DV2 or developmentally vulnerable for each AEDC domain. We specified a series of models to adjust the results, i) Model 0 was unadjusted; ii) Model 1 adjusted for child’s sex and age at the time of AEDC completion; iii) Model 2 additionally adjusted for potentially confounding pregnancy- and birth-related variables (maternal smoking status during pregnancy, preterm birth, small for gestational age, parity, and maternal age at time of child’s birth); and, iv) Model 3 additionally adjusted for sociodemographic variables (child speaks a language other than English at home, ethnicity of child, preschool attendance, maternal marital status at the time of child’s birth, maternal and paternal occupation status, ARIA category, and IRSD category. Risk ratio (RR) and associated 95% confidence intervals (CIs) were estimated for developmental vulnerability within each post-birth IPI category compared to the reference category (post-birth IPIs of 18–24 months). All statistical analyses were conducted in SAS v9.4 [34].

### Ethics statement

This study was conducted in accordance with the Australian National Health and Medical Research Council’s National Statement on Ethical Conduct in Human Research [35]. AEDC data collection occurs under passive consent [36]; thus, written informed consent was not required. A waiver of consent for the use of participant data was granted by the WA Department of Health Human Research Ethics Committee (2016/51) and the University of Western Australia Human Research Ethics Committee (RA/4/20/4776).

## Results

The mean post-birth IPI was 22.8 months (standard deviation [SD], 13.4 months). Overall, a total of 13,902 (43.0%) children had a short post-birth IPI (<18 months), whilst 12,799 (39.6%) of children had a long post-birth IPI (≥24 months: Table 1).

## Associations between post-birth IPIs and developmental vulnerability

**Fig 2:**
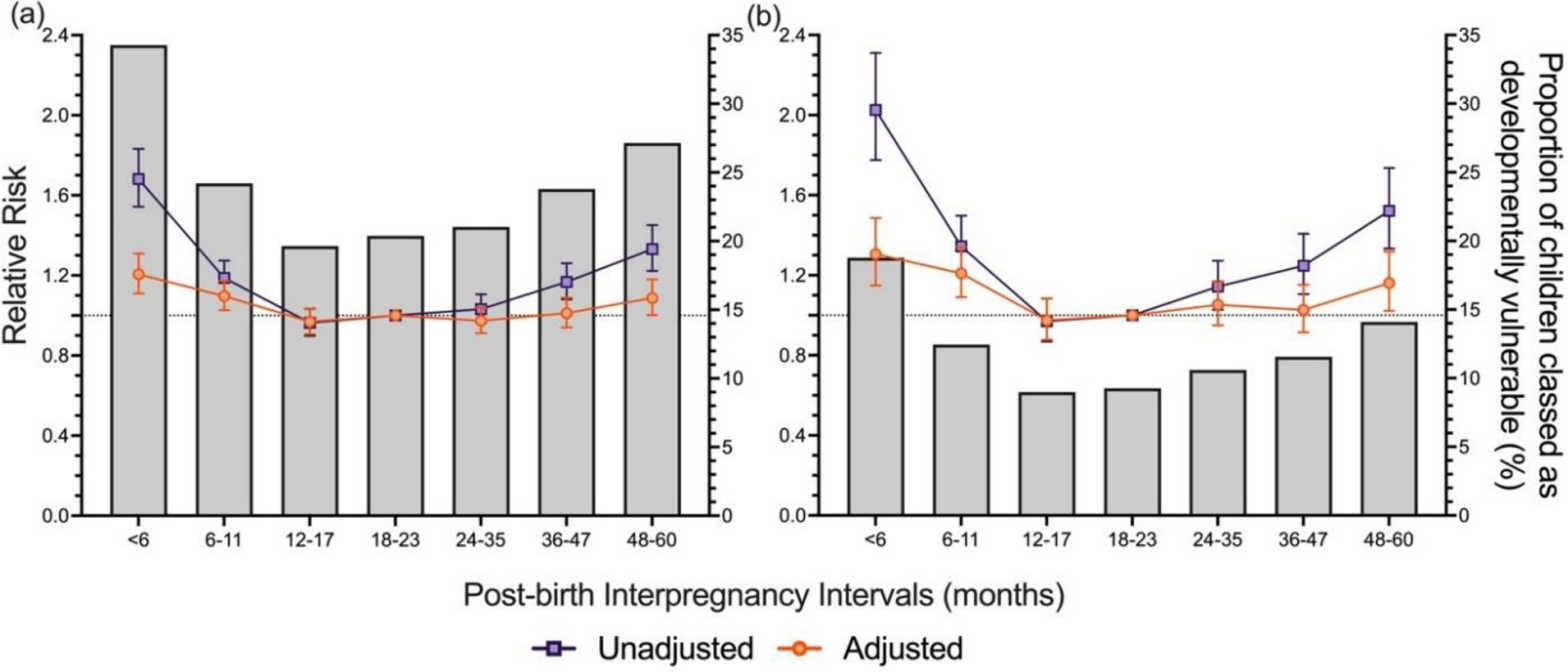
Unadjusted and adjusted relative risk for the association between developmental vulnerability on the Australian Early Developmental Census (AEDC) and post-birth interpregnancy intervals (IPIs). Post-birth IPI was defined as the time between the birth of the child in the cohort and the start of pregnancy of their next youngest sibling (birth date of cohort child minus gestational age of the subsequent child, measured in completed weeks of gestation). The proportion of the study population classified as developmentally vulnerable (a) on one or more AEDC domains, and (b) on two or more AEDC domains, overlayed with the relative risk for each outcome. Developmental vulnerability was defined as scores in the bottom decile, based on the 2009 AEDC cut-offs. Adjusted model based on pooled analysis from 20 imputed datasets, controlling for; the sex of child and age of the child at the time of AEDC completion, maternal smoking status during pregnancy, preterm birth, small for gestational age, parity, maternal age at the time of child’s birth, the child speaks a language other than English at home, ethnicity of the child, preschool attendance, maternal marital status at the time of child’s birth, maternal and paternal occupation status, Accessibility and Remoteness Index of Australia category, Index of Relative Socioeconomic Disadvantage category. All data is presented with 95% confidence intervals: modified Poisson regression.

Of the total cohort, 7,277 children (22.5%) were classified as DV1, and 3,577 children (11.4%) were classified as DV2. The proportion and relative risk of children classified as DV1 and DV2 followed a reverse J-shaped distribution across post-birth IPIs; children with the shortest post-birth IPIs had the greatest proportion and risk of developmental vulnerability (Fig 2).

Children with short post-birth IPIs of <6 months were at an increased risk of being classified as DV1 (RR: 1.68 95% CI: 1.54–1.83) and DV2 (RR: 2.03, 95% CI: 1.78–2.31), relative to the reference group; post-birth IPIs of 18–23 months (Fig 2: *unadjusted model*). Children with short IPIs of 6–11 months had an increased risk of being classified as DV1 and DV2 compared to children with post-birth IPIs of 18–23 months, 1.19 (95% CI: 1.11–1.27) and 1.34 (95% CI: 1.21–1.50), respectively (Fig 2: *unadjusted model*). Children with long IPIs of 24–35 months had an increased risk of being classified as DV2 only, relative to the reference group (RR: 1.14, 95% CI: 1.03–1.27; Fig 2: *unadjusted model*). Children born with longer post-birth IPIs of 36–47 and 48–60 months were associated with an increased risk, relative to the reference group, of being classified as DV1 and DV2, with a greater risk with increasing post-birth IPI category (Fig 2: *unadjusted model*). Adjustment for the sex and age at AEDC completion resulted in slightly elevated RR of children being classified as DV1 for all post-birth IPI categories and of children being classified as DV2, for all post-birth IPI categories, except 24–35 and 36–47 months (S2 Table: *Model 1*). Adjustment for pregnancy- and birth-related variables attenuated the RR of children being classified as DV1 and DV2 for all post-birth IPI categories (S2 Table: *Model 2*). Furthermore, adjustment for sociodemographic variables further attenuated the RR of children being classified as DV1 and DV2 for all post-birth IPI categories (S2 Table: *Model 3*). In the fully adjusted models, children with short post-birth IPIs of <6 and 6–11 months had an increased RR of being classified as DV1, 1.21 (95% CI: 1.11–1.31) and 1.10 (95% CI: 1.03–1.17), respectively and DV2, 1.31 (95% CI: 1.15–1.49) and 1.21 (95% CI: 1.09–1.34), respectively (Fig 2: *adjusted model*). Children with post-birth IPIs of 48–60 months had an increased risk, relative to the reference group, of being classified as DV1 (RR: 1.09, 95% CI: 1.01–1.18) and DV2 (RR 1.16, 95% CI: 1.02–1.32; Fig 2: *adjusted model*).

### Associations between post-birth IPIs and domain-specific developmental vulnerability

**Fig 3:**
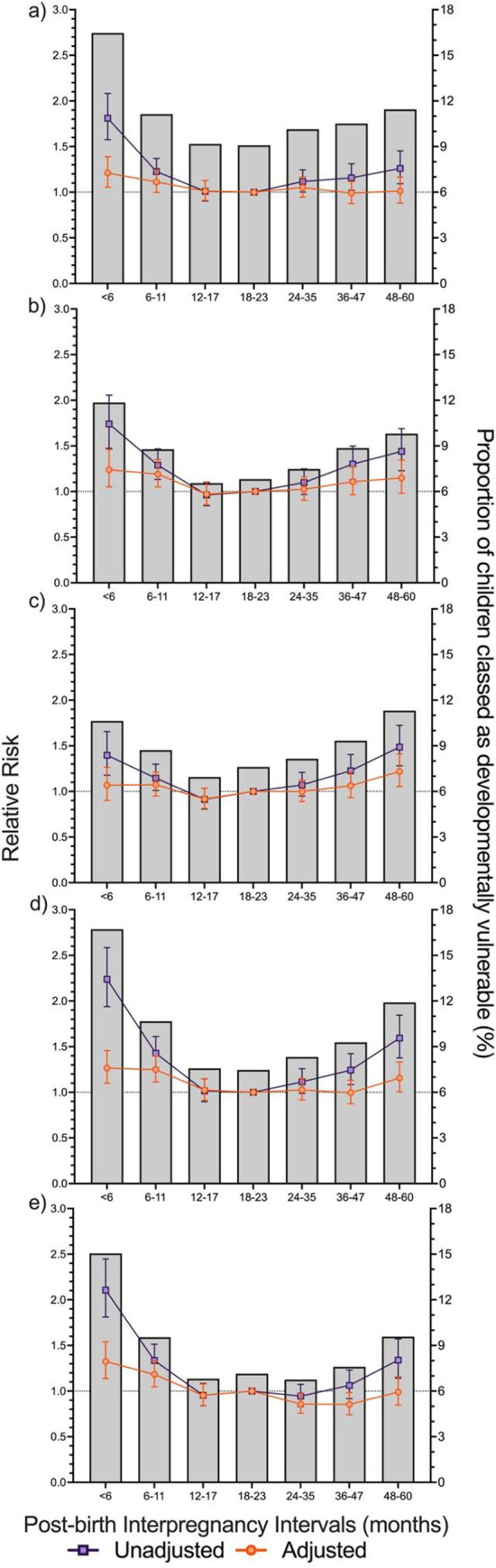
Unadjusted and adjusted relative risk from interaction models for the association between developmental vulnerability for each of the five Australian Early Developmental Census (AEDC) domains. a) Physical Health and Wellbeing, b) Social Competence, c) Emotional Maturity, d) Language and Cognitive Skills (school-based), and e) Communication Skills and General Knowledge and post-birth interpregnancy intervals (IPIs). Post-birth IPI was defined as the time between the birth of the child in the cohort and the start of pregnancy of their next youngest sibling (birth date of cohort child minus gestational age of the subsequent child, measured in completed weeks of gestation). The proportion of the study population classified as developmentally vulnerable overlayed with the relative risk of developmental vulnerability for each outcome. Developmental vulnerability was defined as scores in the bottom decile, based on the 2009 AEDC cut-offs. Adjusted model based on pooled analysis from 20 imputed datasets, controlling for; sex of child and age of the child at the time of AEDC completion, maternal smoking status during pregnancy, preterm birth, small for gestational age, parity, maternal age at the time of child’s birth, the child speaks a language other than English at home, ethnicity of child, preschool attendance, maternal marital status at the time of child’s birth, maternal and paternal occupation status, Accessibility and Remoteness Index of Australia category, Index of Relative Socioeconomic Disadvantage category. All data is presented with 95% confidence intervals: modified Poisson Regression.

Results were broadly consistent with findings for the aggregate measures of developmental vulnerability (Fig 3). Adjustment for pregnancy- and birth-related variables attenuated the RR of children being classified as developmentally vulnerable for all five AEDC domains for all post-birth IPI categories (S3 Table: *Model 2*). Further adjustment for sociodemographic variables further attenuated the RR as developmentally vulnerable for all five AEDC domains for all post-birth IPI categories (S3 Table: *Model 3*). Post-birth IPIs of 48–60 months for the domains of Emotional Maturity (RR: 1.22, 95% CI: 1.05–1.41) and Language and Cognitive Skills (RR: 1.16, 95% CI: 1.01–1.33) (school-based), only (Fig 3).

### Sensitivity analysis

Sensitivity analysis revealed that the overall associations between post-birth IPIs and developmental vulnerability at age five were not substantially different between complete cases and the imputed cases (S1 Table).

## Discussion

We found the risk of developmental vulnerability for all five AEDC domains, and the aggregated measures (DV1 and DV2) of child development followed a reverse J-shaped relationship with respect to post-birth IPIs. The risk of developmental vulnerability was highest for children with a post-birth IPI of <6 and 6–11 months. Our results provide support for the theoretical assumptions of the negative effect of sibling births spaced closely together (i.e., siblings with short post-birth IPIs) on older siblings. Furthermore, we reported that for long post-birth IPIs, the risk of developmental vulnerability generally increased–the risk of developmental vulnerability was statistically significant for children with post-birth IPIs of 48–60 months for DV1, DV2 and for the domains of Emotional Maturity and Language and Cognitive Skills (school-based). Adjustment for pregnancy, birth and sociodemographic variables accounted for a substantial proportion of the risk associated with sub-optimal post-birth IPI durations.

Only a handful of studies have examined the associations between pregnancy spacing and school readiness or academic performance [37–41]. One of the earliest studies investigated the associations between preceding and succeeding birth intervals and school performance of 560 nine-year-old Singaporean children [40]. This study reported insufficient evidence for an association between child development outcomes, as measured by the Raven’s Progressive Matrices, and increasing succeeding birth intervals [40]. However, a small cross-sectional study of 536 Saudi boys, aged 9–10 years, examining the association between succeeding birth intervals and school performance, reported that school performance increased as succeeding birth intervals increased (OR 1.1, 95% CI 1.0–1.2) [37]. Similarly, another cross-sectional study of Saudi boys of the same age group reported that more children born after a long birth interval of >35 months were classified as average or above-average according to the Standard Progressive Raven’s Matrices test compared to children born after a short birth interval of <19 months (p<0.036) [38]. Furthermore, this study reported that scores on the Standard Progressive Matrices Test increased as succeeding birth interval increased; however, this finding was statistically insignificant [38]. Variations in the findings of these studies may be attributed to the relatively small sample sizes and differences in the definition of exposure categories. Despite reporting mixed findings, when taken together, results of the Singaporean and both Saudi studies provide evidence to suggest that short succeeding birth intervals are associated with developmental vulnerabilities in school-aged children. Our findings also indicate that short post-birth IPIs of <12 months are associated with an increased risk of developmental vulnerability in children in their first year of full-time school. Our results build on the current evidence to suggest that increased spacing between siblings may allow for greater levels of investment in older children.

Our results are further supported by the findings of a larger cohort study of approximately 5,000 sibling pairs, aged between 14 and 22 years, from a representative US sample that aimed to assess the relationship between birth spacing and educational attainment [41]. The study reported that increasing birth intervals between the first and second child were associated with improved Peabody Individual Achievement Test scores for the first child [41]. Thus, short post-birth IPIs may be associated with lower levels of resource investment in the older sibling or greater resource constraints within families. This dilution of resources between closely spaced siblings may contribute to an increased risk of developmental vulnerabilities observed in children during the early childhood period.

Albeit generally statistically non-significant, we observed that post-birth IPIs longer than the reference category were associated with an increased risk of developmental vulnerability, with a greater risk for each increasing post-birth IPI category for each AEDC domain. Specifically, we reported that post-birth IPIs of 48–60 months were associated with an increased risk of developmental vulnerability for the domains of Emotional Maturity and Language and Cognitive Skills (school-based). These results provide some preliminary evidence to suggest that perhaps any potential benefits of sibling interactions associated with *the resource augmentation hypothesis*, or the pedagogic experience associated with the *no-one-to-teach hypothesis* may be lost due to the interval between siblings being too large to be beneficial. It should be noted that a wide range of biological factors [42, 43], including sexual activity, fertility levels [44], the use of contraception, medical conditions, pregnancy complications and outcomes [45], and breastfeeding duration, can also govern the intervals between pregnancies. Thus, future studies with larger sample sizes for longer post-birth IPI categories are required to accurately assess the impact of long post-birth IPIs on child development outcomes.

### Limitations

Other important social risk factors, including parenting experience or technique, stability and quality of housing, the total number of people residing within a household, and availability of learning resources within the household, could not be controlled (i.e., proportional of household income spent on learning resources). Total household size and household composition may have both positive and negative effects on household recourse–multigenerational households may be beneficial to improving child development outcomes via grandparental investment [46]. Likewise, smaller household sizes or dependency of a member of a given household (due to old age or disabilities) may result in a redistribution away from children and/or a reduction in total household resources. Given that these factors may affect household resources, future studies should aim to control for such factors. Administrative records do not include pregnancies ending prior to 20 weeks of gestations; thus, we were unable to identify and account for the effect of miscarriages. Likewise, we did not have information as to whether the cohort or the subsequent pregnancies were planned or unplanned.

## Conclusions

Very short post-birth IPIs of <6 and 6–11 months were associated with an increased risk of children being classified as developmentally vulnerable on aggregate measures (DV1 and DV2) and four of the five AEDC domains. Long post-birth IPIs of 48–60 months were also associated with an increased risk of developmental vulnerability on the aggregate measures. The results of this study expand on the current evidence base and suggest that associations between birth spacing and early child development outcomes are observable at school starting age. Although further studies are required, optimising birth spacing between siblings may be a potential means to improve child development outcomes.

## Supporting information

S1 Table Comparison of results from the complete case dataset (n = 32,324) versus imputed dataset (n = 27,265) to estimate the adjusted Relative Risk (RR)^a^ between developmental vulnerability for on Australian Early Development Census (AEDC) domains and Post-birth Interpregnancy Intervals (IPIs). (DOCX)

S2 Table Relative risk (RR)^a^ from interaction models for the association between developmental vulnerability on the Australian Early Developmental Census (AEDC) and Post-birth Interpregnancy Intervals (IPIs). (DOCX)

S3 Table Relative risk (RR)^a^ from interaction models for the association between developmental vulnerability on the Australian Early Developmental Census (AEDC) domains and Post-birth Interpregnancy Intervals (IPIs). (DOCX)

## Abbreviations

AEDC: Australian Early Development Census
ARIA: Accessibility and Remoteness Index of Australia
AUSEI06: Australian Socioeconomic Index 2006
CI: Confidence Interval
IPI: Interpregnancy Interval
IRSD: Index of Relative Socioeconomic Disadvantage
RR: Relative Risk
