# Supplementary material for: Is early childhood development impeded by the birth timing of the younger sibling?
Source: PLoS One. 2022 May 10;17(5):e0268325. doi: 10.1371/journal.pone.0268325 (PMC9089893; doi:10.1371/journal.pone.0268325)
Supplement: S1 Table — (DOCX) [file pone.0268325.s001.docx]

**S1 Table.** Comparison of results from the complete case dataset (n=32,324) versus imputed dataset (n=27,265) to estimate the adjusted Relative Risk (RR)^a^ between developmental vulnerability for on Australian Early Development Census (AEDC) domains and post-birth Interpregnancy Intervals (IPIs).

| **Post-Birth Interpregnancy Interval**  (months) | **AEDC Domain**  RR [95% CI]^a^ | | | |
| --- | --- | --- | --- | --- |
|  | **Developmentally vulnerable on one or more AEDC domain** | | **Developmentally vulnerable on one or more AEDC domain** | |
|  | **Complete Cases**  *n* = 5,392 (19.8)^b^ | **Imputed Cases^c^**  *n* = 7,277 (22.5) | **Complete Cases**  *n* = 2,504 (9.2) | **Imputed Cases**  *n* = 3,577 (11.4) |
| <6 | **1.21 [1.09-1.34]** | **1.21 [1.11-1.31]** | **1.30 [1.10-1.53]** | **1.31 [1.15-1.49]** |
| 6-11 | **1.12 [1.04-1.22]** | **1.10 [1.03-1.17]** | **1.27 [1.12-1.44]** | **1.21 [1.09-1.34]** |
| 12-17 | 0.95 [0.88-1.03] | 0.97 [0.91-1.04] | 0.98 [0.86-1.12] | 0.98 [0.88-1.08] |
| 18-23 | 1 [ref] | 1 [ref] | 1 [ref] | 1 [ref] |
| 24-35 | 0.98 [0.91-1.06] | 0.97 [0.91-1.04] | 0.98 [0.88-1.08] | 1.05 [0.95-1.17] |
| 36-47 | 0.99 [0.91-1.09] | 1.01 [0.94-1.09] | 0.99 [0.88-1.12] | 1.03 [0.91-1.15] |
| 48-60 | **1.12 [1.01-1.23]** | **1.09 [1.01-1.18]** | **1.16 [1.01-1.33]** | **1.16 [1.02-1.32]** |

^a^Data presented as Relative Risk [95% Confidence Intervals]; modified Poisson regression.

^b^Number of children (percentage of children) classified as developmentally vulnerable.

^c^Imputed results based on pooled analysis from 20 imputed datasets.

Adjusted model based on pooled analysis from 20 imputed datasets, controlling for; sex of child and age of child at time of AEDC completion, maternal smoking status during pregnancy, preterm birth, small for gestational age, parity, maternal age at time of child’s birth, child speaks language other than English at home, ethnicity of child, preschool attendance, maternal marital status at time of child’s birth, maternal and paternal occupation status, Accessibility and Remoteness Index of Australia category, Index of Relative Socioeconomic Disadvantage category.
