# Supplementary material for: Is early childhood development impeded by the birth timing of the younger sibling?
Source: PLoS One. 2022 May 10;17(5):e0268325. doi: 10.1371/journal.pone.0268325 (PMC9089893; doi:10.1371/journal.pone.0268325)
Supplement: S3 Table — (DOCX) [file pone.0268325.s003.docx]

| **AEDC domain and model** | | **Post-birth Interpregnancy Interval (months)**  RR [95% CI]^a^ | | | | | | |
| --- | --- | --- | --- | --- | --- | --- | --- | --- |
|  |  | **<6** | **6-11** | **12-17** | **18-23** | **24-35** | **36-47** | **48-60** |
| **Physical Health & Wellbeing** | n (%)^b^ | 257 (16.5) | 594 (11.1) | 643 (9.2) | 511 (9.1) | 697 (10.1) | 393 (10.5) | 251 (11.5) |
|  | Unadjusted | **1.81 [1.58-2.08]** | **1.23 [1.10-1.37]** | 1.01 [0.90-1.13] | 1 [ref] | 1.12 [1.00-1.24] | **1.16 [1.02-1.31]** | **1.26 [1.09-1.45]** |
|  | Model 1^c^ | **1.83 [1.60-2.10]** | **1.23 [1.10-1.37]** | 1.02 [0.91-1.13] | 1 [ref] | 1.11 [1.00-1.24] | **1.15 [1.02-1.31]** | **1.26 [1.10-1.46]** |
|  | Model 2^d^ | **1.29 [1.13-1.48]** | **1.15 [1.03-1.28]** | 1.03 [0.92-1.15] | 1 [ref] | 1.07 [0.96-1.19] | 1.02 [0.90-1.15] | 1.05 [0.91-1.21] |
|  | Model 3^e^ | **1.21 [1.06-1.39]** | 1.11 [1.00-1.24] | 1.01 [0.91-1.13] | 1 [ref] | 1.05 [0.95-1.17] | 0.99 [0.88-1.12] | 1.01 [0.88-1.17] |
| **Social Competence** | n (%) | 185 (11.9) | 468 (8.8) | 459 (6.5) | 383 (6.8) | 514 (7.5) | 331 (8.9) | 215 (9.8) |
|  | Unadjusted | **1.74 [1.47-2.05]** | **1.29 [1.13-1.47]** | 0.96 [0.84-1.10] | 1 [ref] | 1.10 [0.97-1.25] | **1.30 [1.13-1.50]** | **1.44 [1.23-1.69]** |
|  | Model 1 | **1.77 [1.50-2.09]** | **1.29 [1.13-1.46]** | 0.97 [0.85-1.11] | 1 [ref] | 1.09 [0.96-1.24] | **1.29 [1.13-1.49]** | **1.44 [1.23-1.69]** |
|  | Model 2 | **1.31 [1.11-1.55]** | **1.23 [1.08-1.39]** | 0.98 [0.87-1.12] | 1 [ref] | 1.05 [0.92-1.19] | 1.15 [1.00-1.32] | **1.21 [1.03-1.41]** |
|  | Model 3 | **1.24 [1.05-1.46]** | **1.19 [1.05-1.35]** | 0.97 [0.85-1.10] | 1 [ref] | 1.02 [0.90-1.16] | 1.11 [0.96-1.27] | 1.15 [0.98-1.34] |
| **Emotional Maturity** | n (%) | 166 (10.6) | 465 (8.7) | 487 (6.9) | 428 (7.6) | 560 (8.2) | 349 (9.3) | 248 (11.3) |
|  | Unadjusted | **1.40 [1.18-1.66]** | **1.15 [1.01-1.30]** | 0.91 [0.81-1.03] | 1 [ref] | 1.07 [0.95-1.21] | **1.23 [1.07-1.40]** | **1.49 [1.28-1.72]** |
|  | Model 1 | **1.43 [1.21-1.69]** | **1.14 [1.01-1.29]** | 0.92 [0.82-1.05] | 1 [ref] | 1.06 [0.94-1.19] | **1.22 [1.07-1.39]** | **1.49 [1.29-1.73]** |
|  | Model 2 | 1.12 [0.95-1.33] | 1.10 [0.97-1.24] | 0.93 [0.82-1.05] | 1 [ref] | 1.02 [0.91-1.15] | 1.11 [0.97-1.26] | **1.28 [1.11-1.48]** |
|  | Model 3 | 1.07 [0.90-1.26] | 1.07 [0.95-1.21] | 0.92 [0.81-1.04] | 1 [ref] | 1.00 [0.89-1.12] | 1.06 [0.93-1.21] | **1.22 [1.05-1.41]** |
| **Language and Cognitive Skills (school-based)** | n (%) | 261 (16.7) | 569 (10.7) | 531 (7.6) | 420 (7.5) | 572 (8.3) | 347 (9.3) | 261 (11.9) |
|  | Unadjusted | **2.24 [1.94-2.59]** | **1.43 [1.27-1.61]** | 1.01 [0.90-1.15] | 1 [ref] | 1.11 [0.99-1.26] | **1.24 [1.09-1.42]** | **1.59 [1.38-1.85]** |
|  | Model 1 | **2.26 [1.96-2.61]** | **1.43 [1.27-1.61]** | 1.02 [0.90-1.15] | 1 [ref] | 1.11 [0.98-1.25] | **1.24 [1.08-1.42]** | **1.60 [1.38-1.85]** |
|  | Model 2 | **1.38 [1.20-1.60]** | **1.29 [1.15-1.45]** | 1.04 [0.92-1.17] | 1 [ref] | 1.04 [0.93-1.17] | 1.02 [0.90-1.16] | **1.21 [1.05-1.40]** |
|  | Model 3 | **1.27 [1.10-1.46]** | **1.25 [1.11-1.40]** | 1.02 [0.91-1.15] | 1 [ref] | 1.03 [0.92-1.15] | 0.99 [0.87-1.13] | **1.16 [1.01-1.33]** |
| **Communication Skills and General Knowledge** | n (%) | 235 (15.1) | 509 (9.5) | 478 (6.8) | 402 (7.1) | 464 (6.8) | 284 (7.6) | 210 (9.6) |
|  | Unadjusted | **2.11 [1.81-2.45]** | **1.33 [1.18-1.51]** | 0.95 [0.84-1.08] | 1 [ref] | 0.94 [0.83-1.07] | 1.06 [0.92-1.23] | **1.34 [1.14-1.57]** |
|  | Model 1 | **2.13 [1.83-2.48]** | **1.34 [1.18-1.51]** | 0.96 [0.85-1.09] | 1 [ref] | 0.94 [0.82-1.07] | 1.06 [0.91-1.22] | **1.34 [1.15-1.57]** |
|  | Model 2 | **1.45 [1.25-1.69]** | **1.23 [1.09-1.40]** | 0.97 [0.86-1.10] | 1 [ref] | 0.89 [0.78-1.01] | 0.90 [0.78-1.04] | 1.07 [0.91-1.25] |
|  | Model 3 | **1.32 [1.14-1.54]** | **1.18 [1.05-1.33]** | 0.95 [0.84-1.08] | 1 [ref] | 0.86 [0.76-0.97] | 0.85 [0.74-0.98] | 0.99 [0.85-1.16] |

**S3 Table. Relative risk (RR)^a^ from interaction models for the association between developmental vulnerability on the Australian Early Developmental Census (AEDC) domains and Post-birth Interpregnancy Intervals (IPIs).**

^a^All data is presented as Relative Risk [95% Confidence Intervals], modified Poisson regression.

Adjusted models are based on pooled analysis from 20 imputed datasets.

^b^Number of children (percentage of children) classified as developmentally vulnerable.

^c^Model 1 was adjusted for the sex of the child and age of the child at the time of AEDC completion.

^d^Model 2 was adjusted for all variables as per model 1 and controlled for pregnancy and birth-related variables (maternal smoking status during pregnancy, preterm birth, small for gestational age, parity, and maternal age at time of child’s birth).

^e^Model 3 was adjusted for all variables as per model 2 and controlled for sociodemographic variables (child speaks a language other than English at home, ethnicity of child, preschool attendance, maternal marital status at the time of child’s birth, maternal and paternal occupation status, Accessibility and Remoteness Index of Australia, Index of Relative Socioeconomic Disadvantage).
